# Bioactive glass-collagen/poly (glycolic acid) scaffold nanoparticles exhibit improved biological properties and enhance osteogenic lineage differentiation of mesenchymal stem cells

**DOI:** 10.3389/fbioe.2022.963996

**Published:** 2022-09-07

**Authors:** Shirin Toosi, Hojjat Naderi-Meshkin, Zohreh Esmailzadeh, Ghazal Behravan, Seeram Ramakrishna, Javad Behravan

**Affiliations:** ^1^ Tissue Engineering Research Group (TERG), Department of Anatomy and Cell Biology, School of Medicine, Mashhad University of Medical Sciences, Mashhad, Iran; ^2^ Stem Cells and Regenerative Medicine Research Group, Iranian Academic Center for Education, Culture and Research (ACECR), Mashhad, Iran; ^3^ Faculty of Medicine, Mashhad University of Medical Sciences, Mashhad, Iran; ^4^ Center for Nanofibers and Nanotechnology, Department of Mechanical Engineering, National University of Singapore, Singapore, Singapore; ^5^ Biotechnology Research Center, Pharmaceutical Technology Institute, Mashhad University of Medical Sciences, Mashhad, Iran; ^6^ School of Pharmacy, University of Waterloo, Waterloo, ON, Canada

**Keywords:** bone engineering, scaffold, bioglass, collagen, osteogenic differentiation

## Abstract

Today’s using tissue engineering and suitable scaffolds have got attention to increase healing of non-union bone fractures. In this study, we aimed to prepare and characterize scaffolds with functional and mechanical properties suitable for bone regeneration. Porous scaffolds containing collagen-poly glycolic acid (PGA) blends and various quantities of bioactive glass (BG) 45S5 were fabricated. Scaffolds with different compositions (BG/collagen-PGA ratios (w/w): 0/100; 40/60; 70/30) were characterized for their morphological properties, bioactivity, and mechanical behavior. Then, biocompatibility and osteogenic differentiation potential of the scaffolds were analyzed by seeding mesenchymal stem cells (MSCs). Scaffolds made with collagen-PGA combined with the BG (45S5) were found to have interconnected pores (average pore diameter size 75–115 µm) depending on the percentage of the BG added. Simulated body fluid (SBF) soaking experiments indicated the stability of scaffolds in SBF regardless of their compositions, while the scaffolds retained their highly interconnected structure. The elastic moduli, cell viability, osteogenic differentiation of the BG/collagen-PGA 40/60 and 70/30 scaffolds were superior to the original BG/collagen-PGA (0/100). These results suggest that BG incorporation enhanced the physical stability of our collagen-PGA scaffold previously reported. This new scaffold composition provides a promising platform to be used as a non-toxic scaffold for bone regeneration and tissue engineering.

## Introduction

**FIGURE 1:**
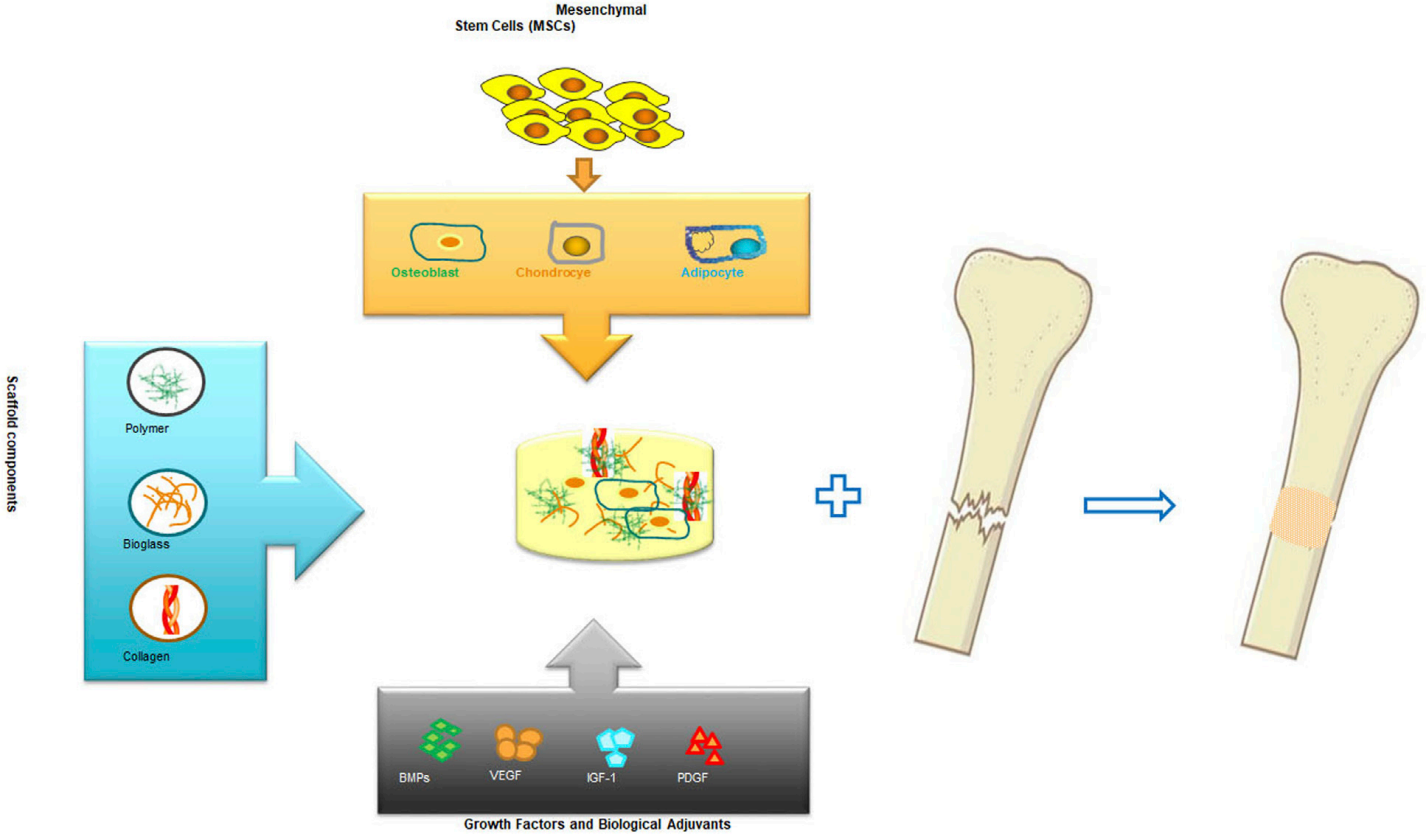
Schematic representation of a bone engineering including its major components.

The science of tissue engineering and regenerative medicine has offered novel approaches for regeneration and repair of tissues and organs which are damaged or lost as a result of trauma, injury or age related conditions (Nerem, 1991). In the most desired condition, a biologically compatible scaffold composite with a well-planned and constructed architecture serves as a temporary structural base for cellular components and guides the differentiation as well as proliferation of tissue forming cell leading to the desired organ or tissue generation. Biomolecules and growth factors can be added into the scaffold, along with the cellular components, to enhance and promote the regulation of cell functions during organ or tissue regeneration (Shea et al., 1999; Babensee et al., 2000; Elisseeff et al., 2001; Mahoney and Saltzman, 2001; Leach et al., 2006; Toosi et al., 2016a; Toosi et al., 2019a) As a general rule, the purpose of this tissue or organ engineering approach is to temporary provide a supporting structure for the cells that contribute to tissue formation enabling them to produce a new tissue with the desired dimensions and shape (Toosi et al., 2016b; Toosi et al., 2018; Toosi et al., 2019b). These investigations have been very fruitful in cellular based bioengineering and regeneration of tissues such as skin (Cooper and Hansbrough, 1991; Hansbrough et al., 1994; Eaglstein and Falanga, 1997; Black et al., 1998), bone (Vacanti et al., 2001; Marcacci et al., 2007; Pishavar et al., 2021)and cartilage (Cao et al., 1997). Figure 1 presents a general schematic bone engineering that includes components used for biocompatible scaffold and cell therapy.

Since the discovery of 45S5 Bioglass^®^ group of materials by Hench (Hench et al., 1971), they have been frequently used as scaffold components for bone repair (Hench et al., 1971; Hench, 1998a; Rahaman et al., 2006; Yunos et al., 2008). BGs are widely recognized for their suitability to support the growth and proliferation of osteoblasts (; Wheeler et al., 1997; Wheeler et al., 1998), and to strongly attach with soft and hard tissues (Hench et al., 1971). Following implantation, BGs undergo very specific types of reactions which lead to the formation of HA (crystalline hydroxyapatite) and ACP (amorphous calcium phosphate) on the glass surface. These specific reactions are shown to be responsible for their strong attachment with the surrounding soft and hard tissue (Fu et al., 2011; Philippart et al., 2015). BGs also release ions that are involved in activation of expression of osteogenic genes (Xynos et al., 2000; Xynos et al., 2001), and play important roles in angiogenesis (Leach et al., 2006; Leu and Leach, 2008). The advantages of BGs include ease of management of their chemical composition and control of the rate of their degradation. Both these properties in addition to other advantages make them attractive scaffold component materials. The chemistry and structure of BGs can be designed using a wide range of options for example by changing either composition, or their environmental and thermal processing. Moreover, scaffolds containing BG may be tailored with variable rates of degradation to meet the optional requirements for bone remodeling and ingrowth.

BGs are mechanically weak. It has been shown that during the process of the fabrication of scaffold, BG can partially crystallize if heated to temperatures above 95°C. Moreover, it has been discovered that in a biological environment and at body temperature, this crystalline phase which is mechanically very strong can transform to a biologically degradable amorphous calcium phosphate (Chen et al., 2006). This transformation property of BG makes it possible to design a scaffold which possesses a combination of biodegradability and mechanical strength at the same time (Boccaccini et al., 2007; Detsch et al., 2015).

In our previous *in vitro* and *in vivo* studies we have shown that a collagen: PGA (0.52 w/w) scaffold exhibited a great potential as a suitable scaffold for bone tissue engineering (Toosi et al., 2016c; Toosi et al., 2018; Toosi et al., 2019b). Here in this study we aimed to examine how 45S5 bioglass affects the biological properties of the collagen-PGA scaffold in favor of bone tissue engineering. In addition, we studied the biological behavior of mesenchymal stem cells derived from bone marrow (BM-MSCs) in the new scaffold prepared from collagen-PGA sponge and BG (45S5).

## Materials and methods

### Collagen sponge fabrication

Type I collagen solution produced by pepsin treatment of porcine tendon (6.33 mg/ml, pH 3.0) in HCl, was obtained from Nitta Gelatin Inc. The fabric of non-woven PGA fiber (20 mm diameter, 0.5 mm thickness, 200–210 g m^−2^) was purchased from Gunze (Kyoto, Japan). BG (45S5) was obtained from MedZist (Tehran, Iran). Fats and oils were removed by immersing the non-woven fabric of PGA in acetone for 1 hour and then a washing procedure was repeated for three times (10 min at 25°C) with double distilled water. In order to obtain the PGA component, tweezers were used to loosen the PGA fiber. PGA fiber (12 mg) was placed inside each well in a 24-well tissue culture plate, and then 1 ml of collagen containing 40 and 70 (w/w) BG was poured over the PGA fiber inside each well. A dehydro-thermal method was used to fabricate collagen sponges including BG and 12 mg of PGA fiber (Toosi et al., 2016c). The obtained scaffold compositions were frozen for 24 h at −20°C resulting in a collagen sponge containing PGA fiber and BG with the following w/w ratios of BG/collagen-PGA: 0/100; 40/60; 70/30 (collagen: PGA 0.52 w/w). A dehydro-thermal procedure (140°C and under 0.1 torr for 12 h) was used for cross linking of the freeze-dried sponge. This procedure of cross-linking is toxicologically more acceptable compared to the alternative chemical crosslinking. Using collagen solution alone, a similar experimental procedure was conducted to prepare a collagen sponge with no incorporation of PGA. Ethylene oxide (40°C) was used for sterilization of the prepared scaffolds.

### Morphological characterization

The infrastructure and appearance of scaffolds [BG/collagen-PGA (w/w): 0/100; 40/60; 70/30] were visualized using a SEM (scanning electron microscopy, Leo, 1450 VP, Germany). A razor blade was used to cut the sponges. The cross sections of the sponges were then coated with gold using E-1010; Hitachi (ion sputterer) 30 s at 5 mA and 50 mTorr. The samples were then visualized using SEM (15 kV). The pictures of the sponge cross sections were used for the calculation of the pore sizes of collagen sponges using the geometric mean values of diameters of the pores (Hu et al., 2008; Mandal and Kundu, 2009; Rahaman et al., 2011).

### Sponge characterization

Mercury intrusion porosimetry (MIP) measures the pore volume and geometry. It works by introducing a liquid with non-wetting properties like mercury into the dry sponge under pressure. First the scaffold composites are placed in a penetrometer and then a high vacuum is applied. When the condition of minimal pressure inside the glass penetrometer is achieved, the sample is surrounded by flowing mercury. The initial volume of mercury in the penetrometer is subtracted from the penetrometer total volume (known) and the obtained value is considered as the volume of the test porous sponge if it were a completely solid sample. Subsequently an increasingly incremental pressure is applied on the mercury reservoir in the stem of the penetrometer at several installments. As the pressure is applied the mercury continuously enters in the pores. To determine the overall porosity of scaffold, the mercury volume (total) which is forced into the test sample is measured. For determination of pore size values, the amount of mercury at each pressure interval is used.

Three samples were sliced from three different composite scaffolds and the porosity values were measured with a mercury porosimeter (LLOYD, INSTRUMENTS, An AMETEK Company). Same batch of scaffolds were used for SEM to facilitate correspondence between the calculated values. Initially, a vacuum (50 mm mercury) was applied on each specimen to completely remove air from the test sample. As described above, mercury was then used to surround the sample and introduced into the pores forcing increasing range of pressures from 0.22 to 30 psi. Prior and following of the introduction of mercury, the sample weights in penetrometer were measured. To obtain the total volume used, the weight difference was divided by the mercury density at ambient temperature. By dividing the value of total volume of the pores by the samples external volume, percent pore volume or porosity was determined. For an estimation of the pore size Washburn equation was used:

$$\mathrm{r=2}s\frac{\cos\left(\theta \right)}{p}$$

where *r* stands for the radius of the pore, *s* is the value for mercury surface tension, Ɵ is the value of the mercury contact angle, and *p* represents the amount of applied pressure. An algorithm that is built in the device automatically calculated the value at various pressure intervals, and after recording all points, the average values were obtained.

### Evaluation of bioactivity

To determine the bioactivity of samples, the prepared porous scaffolds were soaked for various time intervals (two, seven, and 14 days) in 5 ml of SBF pH 7.4 at 37°C (at every 2 days the SBF solution was refreshed). The composition of SBF is very similar to blood plasma of human. SBF has been used in bioactivity assays (*in vitro*) extensively. To conduct compositional analysis and morphological examination, the prepared specimens were taken out from SBF and rinsed intensively with deionized water.

### Mechanical characterization

The compression test performed based on the international standard for compression testing, ISO 604 using a mechanical testing machine (LLOYD, INSTRUMENTS, AMETEK, West Sussex, United Kingdom). The tested cylinder-shaped scaffold samples had a height of approximately one to 1.2 cm with 2 mm diameter as measured with a digital caliper. For every composition, five porous samples were analyzed at ambient temperature. The used crosshead speed was 0.01 mm.S^−1^ and the loads were added until the sample was compressed to almost seventy percent of its initial length. The curves of compressive stress–strain were prepared and for each sample the mean compressive modulus with the relevant standard deviation (SD) value was obtained. The modulus was defined as the value of slope of the stress–strain curve initial linear portion (Wang et al., 2007).

### Cell seeding procedure in the scaffold

BM-MSCs were seeded into the composite scaffolds using the agitated seeding method. Pre-wetted sponges were inserted into a sterile 15 ml tube. Then the cell suspension (1×10^6^ in 0.5 ml) was placed into the tube. The sponge–cell mixture culture was shaken on an orbital shaker (VISION, VS-8480, Korea) at 300 rpm for 6 h. Then the culture was placed for 2 hours in a 24-well plate (5% CO_2_ incubator_)_ at 37°C. 1 ml of DMEM (Sigma-Aldrich, Milan, Italy) supplemented with FBS (15% w/v) and penicillin (100 U.ml^−1^) and 100 μg ml^−1^ streptomycin (GIBCO, Invitrogen, Milan, Italy) was added to each culture.

### MTT assay

Proliferation of MSCs on sponges was determined by the MTT 3-(4, 5-dimethylthiazol-2-yl)-2, 5-diphenyl-2H-tetrazolium bromide (atocell) assay. The sponges were transferred into a new 24-well plate, and 1 ml of MTT solution (0.5 mg/ml) was added to each well. After incubation at 37°C for 4 h in a 5% CO_2_, MTT was taken up by the active cells and reduced in the mitochondria to insoluble purple formazan granules. Subsequently, the medium was discarded and the precipitated formazan was dissolved in DMSO (150 µl/well). The optical density of the solution was evaluated using a microplate spectrophotometer after subtraction of OD 570 nm. The viable cell number was determined using a linear calibration curve between OD and predetermined cell concentration.

### Staining for live/dead differentiation

The whole 3D culture was stained with PI/FDA (propidium iodide/fluorescein diacetate) (FDA; Sigma, F73378 and PI; Sigma, 81,845) to demonstrate the viability of cells. Rinsing procedures (three times with PBS) were then followed and the grafts were incubated for 15 min at 37°C with FDA solution (2 μg ml^−1^). Another rinsing procedure (with PBS for three times) was followed and then the grafts were incubated with PI solution (0.1 mg ml^−1^) at room temperature for 2 min. A final washing step was followed before the screening of the grafts using a fluorescent microscope (Olympus BX51).

### Mesenchymal stem cells derived from bone marrow differentiation towards osteogenic lineage

BM-MSCs were taken from iliac crest bone marrow of healthy donors undergoing bone marrow harvest (approved by medical research ethics committee of Mashhad University of Medical Sciences (MUMS) project number 951244). To study the osteogenic lineage differentiation of the BM-MSCs, the bone osteocalcin content and ALP (intracellular alkaline phosphatase) activity were evaluated. The cells seeded on BG/collagen-PGA sponge cultured with a similar method as described above. DMEM (low glucose) was supplemented with FBS (10%), ascorbic acid (50 mg ml^−1^), dexamethasone (10 nM) and 10 mM β-glycerophosphate (differentiation medium) (Toosi et al., 2017). For control experiments DMEM (low glucose) with FBS (10%) was used. ALP activity was assayed with an ALP assay kit (Lot. No. APF; Sigma, Missouri, United States). The cultured sponges were washed three times with PBS, then minced with scissors, and finally homogenized in a lysis buffer consisting of 0.2% triton X-100 (w/v), Tris–HCl (10 mM), MgCl_2_ (1 mM) and pH 7.5. Two ml of the sample lysate was then centrifuged for 10 min (12,000 rpm at 4°C). Then supernatant ALP activity was measured using the substrate *p*-nitrophenyl phosphate. In order to test for calcium deposition, the cultured grafts were washed (3X with PBS). An aqueous solution of trypsin 0.05% (w/v) and 1 mM EDTA (in 0.1 M PBS), pH 7.4 (GIBCO, Invitrogen, Milan, Italy) was then used to detach BM-MSCs from the cultured scaffold. Attached BM-MSCs to the plates were fixed with formaldehyde 3% (w/v) for 10 min at ambient temperature. The specimens were then placed in alizarin red stain (pH 4, room temperature). The samples were then washed with PBS (3X) for 10 min to visualize the differentiated cells. The stain (alizarin red) was then removed and an equal volume of PBS was added. An Eyepiece camera (AM4023; Dino-lab digital microscope, United States) was used to take pictures.

### Statistical analysis

All measured values are shown as mean ± SD (standard deviation). ANOVA (single-factor analysis of variance) was used for statistical analysis. A calculated value of *p* < 0.05 represents a statistically significant value.

## Results

### Porosity and morphological analysis

Morphological and porosity values of collagen scaffold reinforced with PGA and different amounts of BG are shown in Table 1. Our results indicate that the porosity of collagen-PGA scaffolds was decreased by incorporation of BG to a minimum level at incorporation rate of 40% (w/w) BG. This could be due to the filling of the pores by adding BG and increased interconnectivity of pores.

**TABLE 1:** Characterization of porosity of the collagen/poly (glycolic acid) (PGA) blends containing different amounts of a bioactive glass (45S5).

Collagen/fiber ratio (w/w)	45S5/PGA-collagen 0/100	45S5/PGA-collagen 40/60	45S5/PGA-collagen 70/30
Average pore size (µm)	72.5	113.5	112.5
Porosity (%)	91.39	62.4	73.6

**FIGURE 2:**
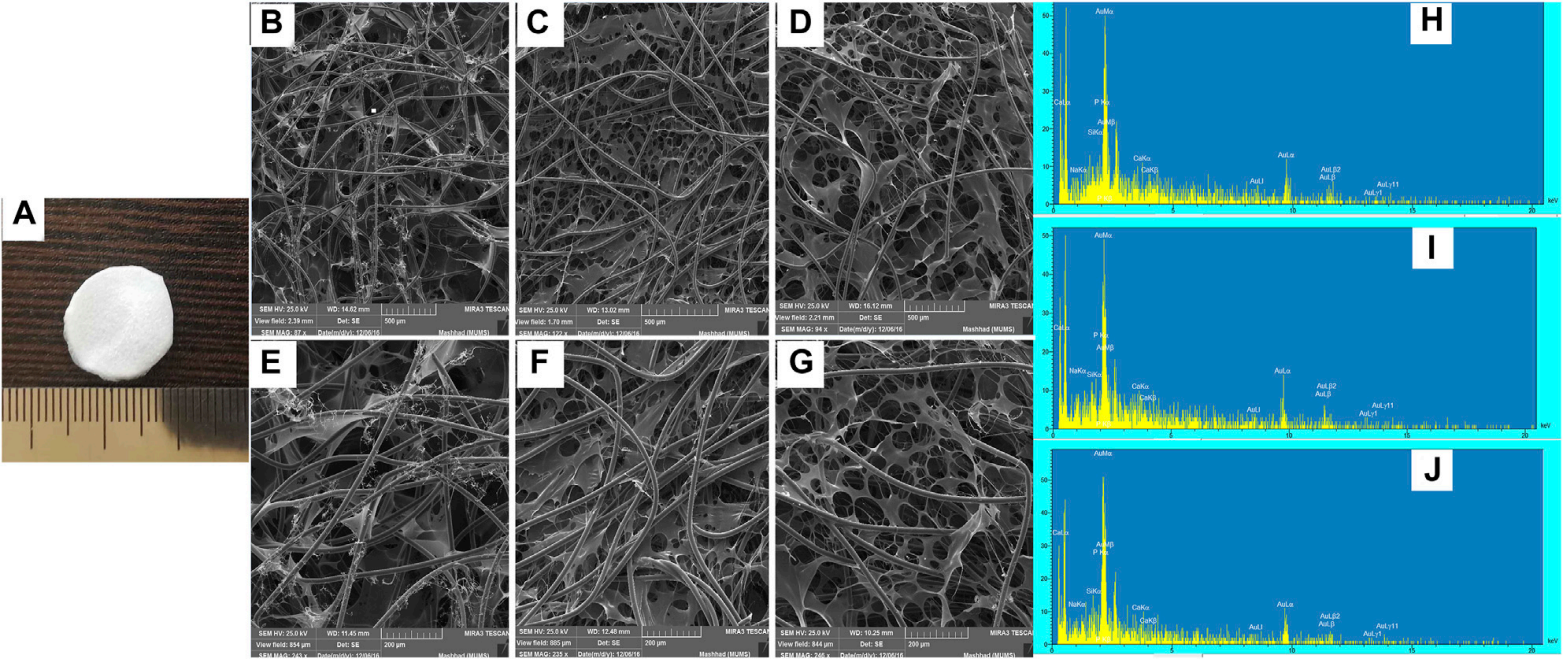
Frame structure, SEM micrographs and EDS spectra of BG-Collagen/PGA scaffolds: Physical picture of BG-collagen/PGA scaffold **(A)**. Section of **(B,E,H)** 5S5/PGA and collagen 0/100, **(C,F,I)** 45S5/PGA and collagen 40/60, **(D,G,J)** 45S5/PGA and collagen 70/30. The dispersion of BG nanoparticles in PGA matrix can be seen **(C,F,D,G)**.

**FIGURE 3:**
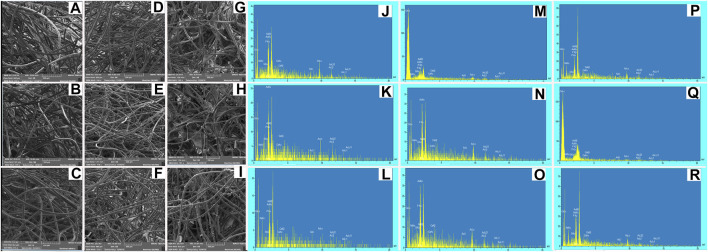
SEM micrographs and EDS spectra of BG-Collagen/PGA scaffolds after immersion in SBF for different intervals: after 2 days **(A,J)** 45S5/PGA and collagen 0/100, **(B,K)** 45S5/PGA and collagen 40/60, **(C,L)** 45S5/PGA and collagen 70/30, after 7 days **(D,M)** 45S5/PGA and collagen 0/100, **(E,N)** 45S5/PGA and collagen 40/60, **(F,O)** 45S5/PGA and collagen 70/30, and after 14 days **(G,P)** 45S5/PGA and collagen 0/100, **(H,Q)** 45S5/PGA and collagen 40/60, **(I,R)** 45S5/PGA and collagen 70/30 Figure 4. Stress–strain curves: The porous composite scaffolds compressed at a strain of (0%–70%). The cross-head speed was 0.01 mm.s^−1^.

The pore sizes were found to vary from 75 to 115 µm depending on incorporation percentage of the BG. In particular, the average pore size increased with increasing the bioglass portion. In scaffolds prepared with BG (70% w/w), the average pore size was slightly lower than BG, (40 %w/w) incorporation. This average pore size reduction by higher bioglass incorporation could be attributed to the deposition of BG on the collagen-PGA pore walls and therefore, resulting in the partial occupation of the collagen-PGA matrix free void space. This was subsequently confirmed by SEM) Figure 2. A foam-like morphology presenting with a wide distribution of interconnected pores was observed in the prepared scaffolds (Figure 3).

### Bioactivity of the scaffolds

After immersion in SBF, the BG/collagen-PGA composite scaffolds were studied by SEM to investigate the composite surface for its bioactivity properties and the formation of layer of apatite as the interaction between SBF solution and the surface of the composites is expected to cause formation and nucleation of an apatite layer on the scaffold surface.

Figure 3 represents the SEM images of the fractured section of the BG/collagen-PGA scaffold composites after soaking in SBF for two, seven, and 14 days, respectively.

SEM micrographs of the BG/collagen-PGA scaffolds after SBF immersion for different intervals indicated the stability of scaffolds in SBF regardless to the compositions of scaffolds, while they retained their highly interconnected structure.

### Mechanical properties characterization

**FIGURE 4:**
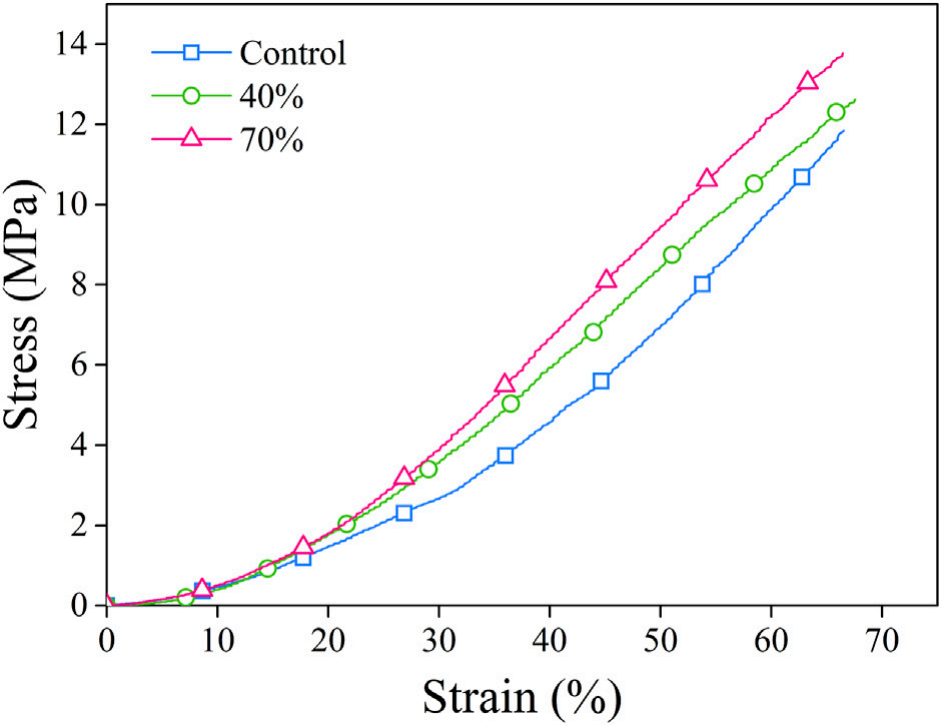
Stress–strain curves. The porous composite scaffolds compressed at a strain of (0%–70%). The cross-head speed was 0.01 mm.s^−1^.

The mechanical characteristics including the compressive strength of the composite sponges were assayed by a mechanical testing machine. By applying a constant compressive load at a fixed speed, the force from stress–strain data was calculated (Figure 4). Table 2 presents the data for stress-strain which was measured for the porous sponges by applying an excessive compression force at BG (0%–70%). Upon compression, the composite scaffolds underwent a procedure of densification and did not present with any fractures in their structure. Three distinctive regions were used for curves classification: collapse plateau, linear elastic, and densification process. The collapse modulus (E′), collapse strength (s^*^), elastic modulus value (E*), and strain (ε^*^) were all obtained from the curves of stress-strain which are presented in Table 2. The elastic moduli of BG/PGA-collagen (40/60) and BG/PGA-collagen (70/30) scaffolds were 1.1 MPa.

**TABLE 2:** Elastic modulus (E*), collapse modulus (E′), collapse strength (σ *) and strain (ε*).

	E* (MPa)	E’ (MPa)	σ* (MPa)	ε* (%)
45S5/PGA-collagen 0/100	1.1 ± 0.2	0.9 ± 0.1	2.3 ± 0.18	27 ± 0.7
45S5/PGA-collagen 40/60	1.1 ± 0.4	1.6 ± 0.23	4.1 ± 0.5	32 ± 0.8
45S5/PGA-collagen 70/30	1.1 ± 0.3	1.6 ± 0.25	4.6 ± 0.6	33 ± 0.7

The composite scaffold mechanical properties strongly depend on the size of inorganic phase component, the aspect ratio, the particle amount and the particle-matrix adhesion. By incorporating of nano/micro particles, the sponge modulus can be increased while the strength characteristics depend on the transfer of stress between the particles and the matrix.

The uniformity of BG particles distribution and their appropriate bonding to the collagen matrix are important contributors and responsible for the approximately two folds increase from 2.3 ± 0.18 MPa to 4.1 ± 0.5 MPa and 4.1 ± 0.6 MPa in collapse strength of the designed composites.

The improved mechanical properties of scaffolds can be linked and attributed to the small size and uniform shape of the incorporated inorganic phase and, also to the properly distributed BG particles within the collagen matrix.

Compared to the PGA-collagen sponge, the inclusion of an inorganic phase resulted in two times increase in the scaffolds mechanical properties. The mechanical specifications of the BG/PGA-collagen 40/60 and 70/30 (w/w) scaffolds were significantly improved due to incorporation of BG. However, the sponge composites obtained by freeze drying with collagen incorporation exhibited very high porosity which lowers the collapse strength and elastic modulus of the resulting composite compared to those of the cancellous bone. Therefore, for non-load bearing bone tissue engineering these composite scaffolds may be used.

### Cell viability

**FIGURE 5:**
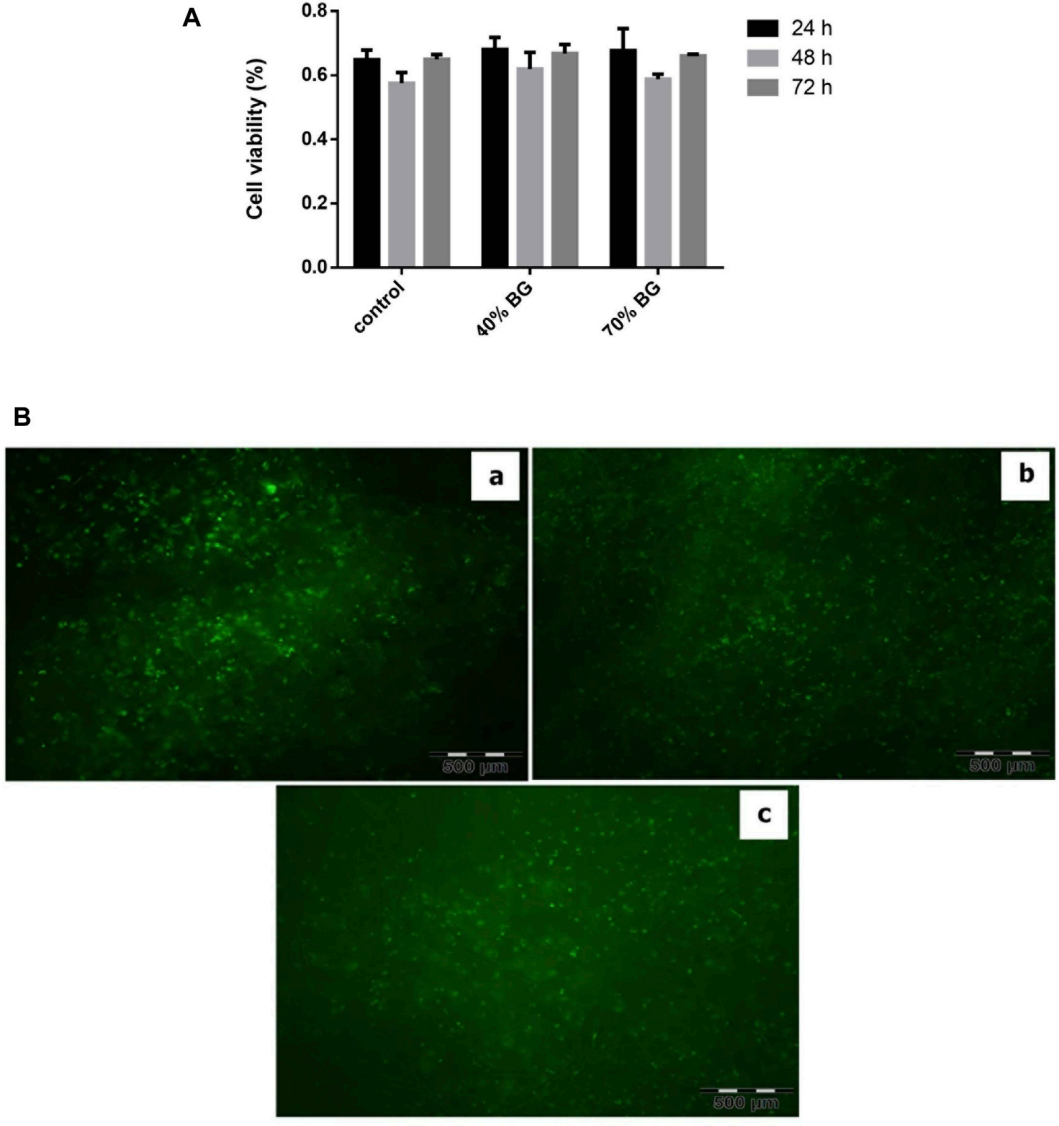
Viability assay of BM-MSCs in the sponges using MTT and PI-FDA staining. **(A)** Cell viability on the BG-collagen/PGA scaffolds. The results were expressed as proliferation of MSCs on scaffolds. Each experiment was repeated independently three times in triplicate tests *p < 0.05; significant against the viability of cells on collagen/PGA scaffold without BG incorporation. **(B)** Cell viability staining with PI/FDA. **(a)** BG/PGA-collagen 0/100, **(b)** BG/PGA-collagen 40/60, **(c)** BG/PGA-collagen 70/30 w/w. The staining presents with viable cells and homogeneous cell distribution within the scaffold (Magnification 10 X). BG containing scaffolds are biocompatible as evidenced by cell attachment, spreading and maintenance of cell viability within the constructs.

MTT assay was used to compare cell viability on BG/collagen-PGA ratios (w/w): 0/100; 40/60; 70/30 (Figure 5A). The results showed no significant different between three groups of scaffolds.

The viability of cells in the scaffold composites was assessed by a staining protocol for live-dead (FDA-PI) after 3-weeks of three-dimensional arrangement of the expanded MSCs (Figure 5). Our findings showed that MSCs were homogeneously distributed in all three different types of scaffolds. The green color staining of cellular components proliferation in collagen-PGA sponges, with different amounts of BG, indicates that the MSCs were viable and consisted of almost round-shaped cells. Our finding supports the fact that the prepared composite scaffolds containing BG were biologically compatible as evidenced by MSCs binding to the scaffold, their spreading, and cell viability maintenance within the composites prepared.

### Differentiation of mesenchymal stem cells derived from bone marrow towards osteogenic lineage.

**FIGURE 6:**
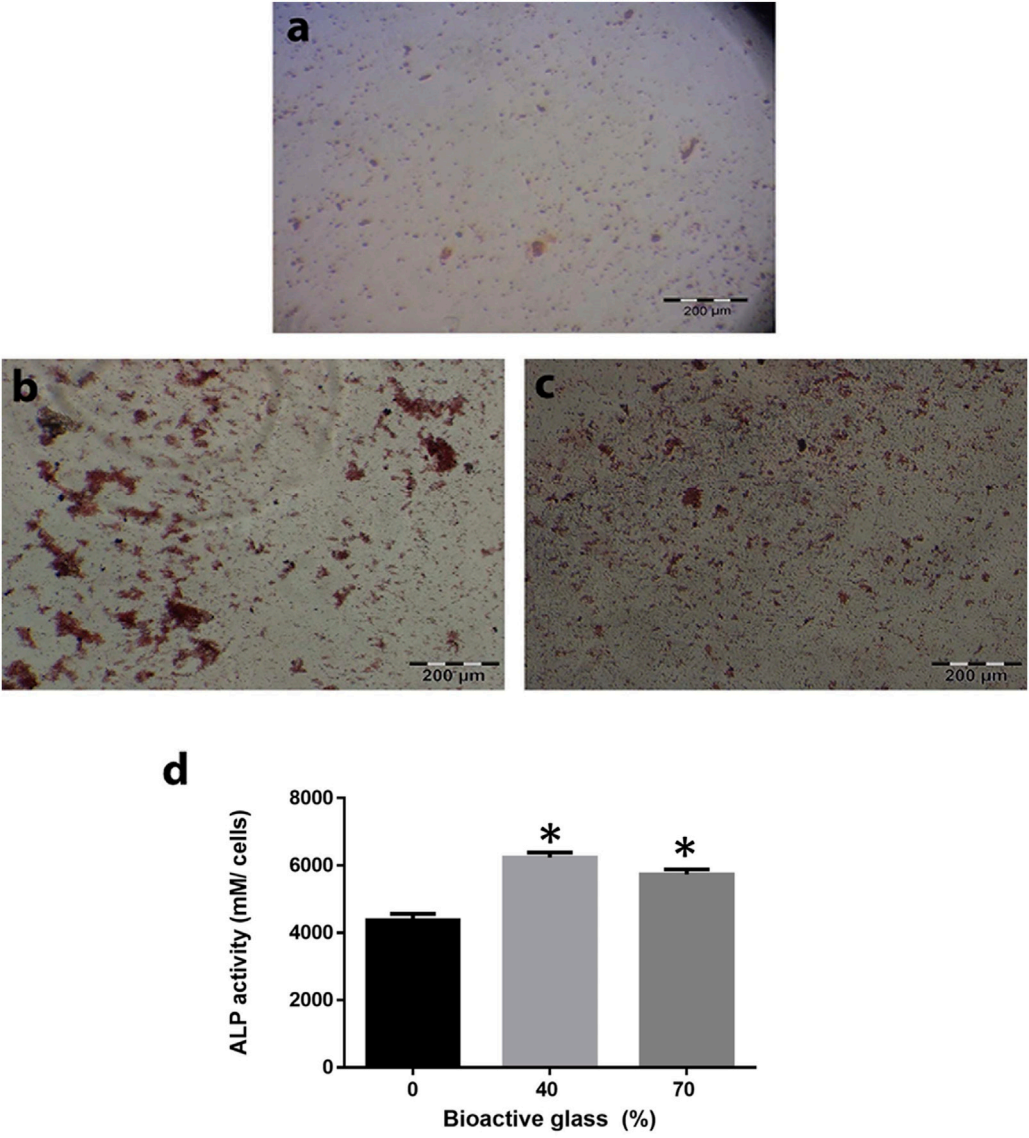
Effect of the different scaffold compositions in induction of differentiation of bone marrow mesenchymal stem cells (BM-MSCs) toward osteoblast lineage. Calcium depositions that produced by BM-MSCs have been shown by alizarin red staining for BG/collagen-PGA: **(A)** 0/100; **(B)** 40/60; **(C)** 70/30 w/w cultured in bone differentiation media after 21 days at 37°C in a 5% CO_2_. Red nodules are shown the ECM deposition as a result of osteogenesis. **(D)** Alkaline phosphatase activity as an index of osteogenesis was also calculated for BM-MSCs culture within all three types of the scaffold. Each experimental procedure was repeated three times. The data are shown as mean ± SD. *p ≤ 0.05 was considered as significant. ALP activity was significantly higher in BG/collagen-PGA: 40/60 and BG/collagen-PGA: 70/30 rather than control BG/collagen-PGA: 0/100.

Figures 6A–C indicates that MSCs differentiated towards osteocytes. Alizarin red staining used to examine calcium deposits of differentiated cells after 21 days. MSCs differentiation to osteoblasts was confirmed by alizarin red staining with increased ALP activity in collagen-PGA sponge with BG incorporation, in comparison to collagen-PGA scaffold without addition of the BG.

Figure 6D shows ALP activity of MSCs cultured on BG/PGA-collagen ratios (w/w): 0/100; 40/60; 70/30. ALP activity of MSC cultured in the bone differentiation medium was high for BG/PGA-collagen 40/60 and 70/30 (w/w), as compared to BG/PGA-collagen 0/100 (w/w). In induction medium, incorporation of BG enhanced ALP activity.

## Discussion

Using *in vivo* and *in vitro* experiments, we have previously shown the desirable specifications and bone healing properties of a collagen-PGA scaffold (Toosi et al., 2016c; Toosi et al., 2018; Toosi et al., 2019b). Here, we aimed to enhance the bioactivity, physical, functional and mechanical properties of our previously reported scaffold designed for bone regeneration application by incorporating bioactive glass 45S5.

Porous scaffolds were prepared using various amounts/ratios of BG to collagen-PGA (w/w): 0/100; 40/60; 70/30). Incorporation of BG into the scaffold composites resulted in scaffolds with average pore sizes of 100 μm (75–115 μm). The porous scaffold structures were analyzed for their stability, mechanical properties and bioactivity supporting MSCs proliferation, metabolic activity and induction of seeded MSCs towards osteogenic lineage differentiation.

One of the critical and most essential requirements for an artificially prepared composite for bone regeneration applications is its ability to form a bioactive bone-like apatite structure on its surface after exposure with a physiological and suitable environment (Toosi et al., 2018). We have now a vast option of biologically active materials such as bioglass and TCP which have been reported to be used for bone repair clinical procedures (Hench, 1998a). These bioactive composites have been shown to be able to bond with bone tissue by means of a bone-mimicking apatite layer which is formed on the bioactive material surface when implanted into the human (patient) body. Further analysis has confirmed that this formed apatite is composed of carbonate-containing HA. This has not been seen at the interface between bone and bio-inert or non-bioactive materials (Kitsugi et al., 1989). There is an *in vitro* oscillating phenomenon consisting of simultaneous mineral component adsorption and resorption processes that is due to non-stable conditions of SBF. The processes of dissolution and precipitation of bone like apatite happens while the bioactive materials are immersed in SBF. As shown in Figure 3 and supported by other data, in SBF soaking experiments our designed scaffolds were stable and retained their interconnected porous structure. Further *in vivo* analysis is required to assess the bone attachment property that is an essential characteristic of an artificial biomaterial used in bone healing. For bone bonding the *in vivo* formation of bone-like apatite consisting of calcium phosphate is an absolute requirement (Hench, 1998b).

While preparing scaffolds for load-bearing bone tissue engineering application, a scientist needs to deal with two conflicting composite requirements, very high mechanical strength and providing a high porosity. In favor of cell proliferation and growth, the scaffold needs to provide a highly porous structure while this property is generally in conflict with another important property of having a high mechanical strength (Gentile et al., 2012). Moreover, the composite scaffold mechanical properties are highly dependent on the aspect ratio, the size of the added mineral phase, the particle quantifications and the particle-matrix adhesion. By incorporation of nano/micro particles, the modulus of a sponge composite can be increased while the strength of the scaffold structure depends on the transfer of stress between the matrix and the particle (Mikael and Nukavarapu, 2011). Compared to our previously designed collagen-PGA scaffold (used as control here), the BG/collagen-PGA at both ratios (40/60 and 70/30) presented with more advanced mechanical properties.

The BG (45S5) has proved to provide enhanced biological activity and its properties in supporting differentiation of stem cells towards osteoblastic lineage have been reported (Gentile et al., 2012; Detsch et al., 2015). Therefore, its incorporation in a scaffold composite results in a highly mineralized bone matrix formation (Xynos et al., 2001; Gentile et al., 2012). It is considered that BG is osteoinductive and osteoconductive. It is able to support formation of new bone growth along the bone–implant interface and even within the implanted sponges away from the interface of bone–implant (Rahaman et al., 2011). Therefore, as we hypothesized, our new scaffolds with the incorporation of BG showed advanced *in vitro* properties and supported the MSCs differentiation to osteoblasts (Figure 5). This latter property may prove to be very critical *in vivo* and will be examined in our future animal studies.

Induction of ALP expression by the bioactive inorganic incorporation and bioglass polymer composites has been shown elsewhere in the literature (Knabe et al., 2005; Yao et al., 2005; Kim et al., 2006). The products released from 45S5 bioglass ionic dissolution are shown to have effects on the regulation of gene expression profile of human osteoblasts in monolayer cultures (Xynos et al., 2001). Tsigkou and co-workers have shown enhanced differentiation and mineralization of fetal osteoblasts and formation of bone nodules induced by a BG conditioned medium in the absence of other supplements for osteogenic differentiation (Yao et al., 2005; Tsigkou et al., 2007). In another study, El-Gendy *et al.*, have shown that both *in vitro* and *in vivo*, BG containing scaffolds are able to enhance human dental pulp stromal cells differentiation to osteoblasts. This strongly supports the potential use of this bioglass for bone tissue engineering for clinical applications (Reilly et al., 2007; El-Gendy et al., 2012). This is in line with our observations in the design of our BG/collagen-PGA scaffolds. Subsequent plans on implementation and design of *in vivo* studies of the scaffolds are underway.

Overall, our findings indicate that BG incorporation would enhance physical stability and biological activities of our previously reported collagen-PGA scaffold towards bone tissue regeneration applications. The novel scaffold composition may provide a basis for additional *in vivo* and *in vitro* studies of bone repair and bone tissue engineering.

## Future works

There is currently an urgent need for methodological studies (design, formulation, and fabrication) of new scaffolds for bone regeneration.

In order to improve the biological and mechanical properties of scaffolds, collagen sponges can be modified by a group of diverse materials. Bioceramics such as BG 45S5, as mineral components are known to be capable to improve the osteointegration, mechanical properties and osteoinduction of composite scaffolds. Moreover, PGA and other polymers enhance the stability and mechanical properties.

One shortcoming in this field is now a lack of reliable and validated experiments *in vivo* to examine the suitability of these scaffolds. Furthermore, there are still challenges in the fabrication of an ideal composite for bone regeneration scaffolds to provide idealistic pore size, mechanical stability and integrity, biocompatibility, osteoinductivity and osteoconductivity. There is also a great need in optimization of scaffolds to provide the required attachment, migration and survival of the cells in volved in bone healing and regeneration.

**FIGURE 7:**
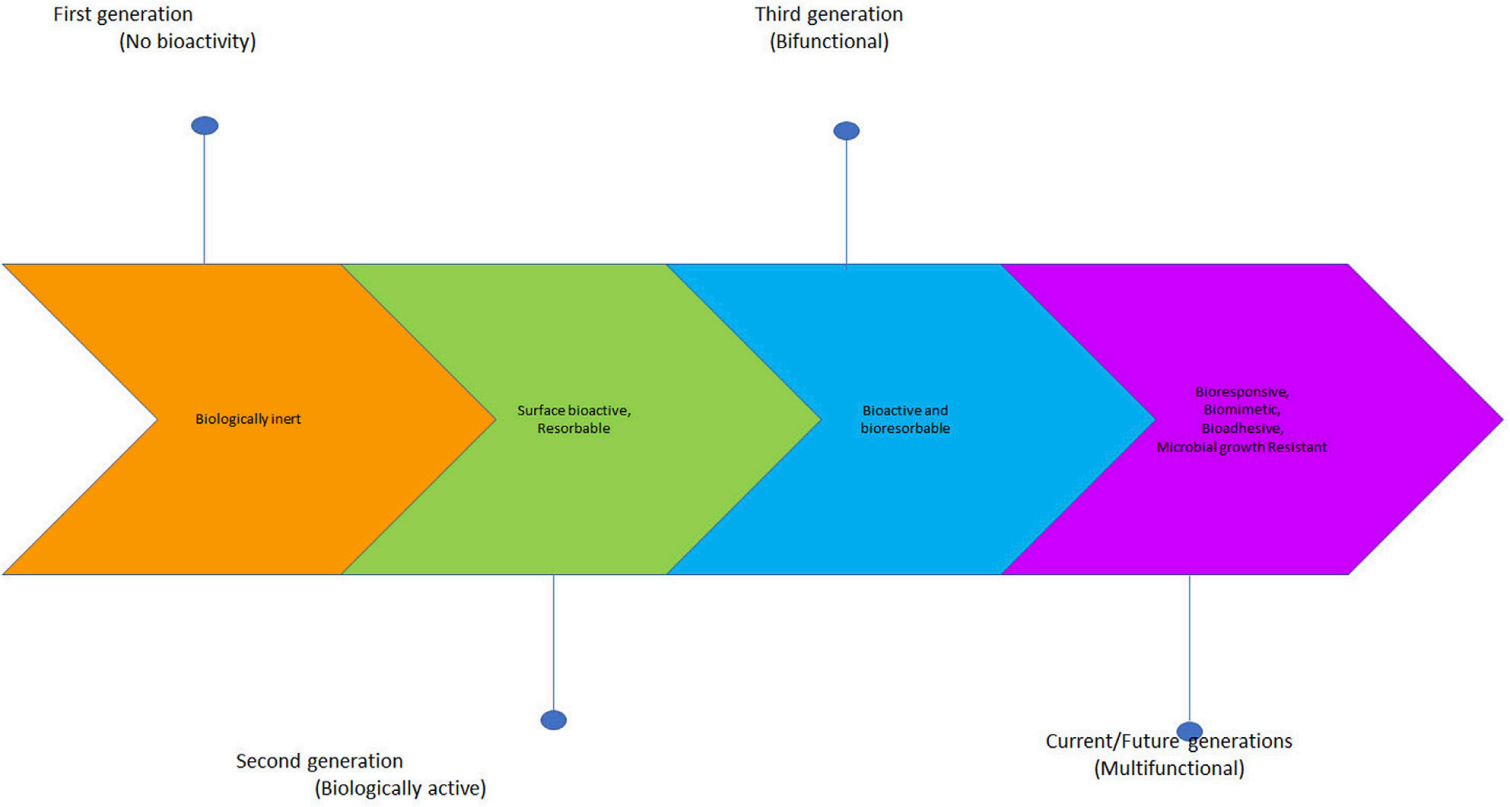
The evolving since of bone regeneration scaffolds.

Following the recent advancements in bone regeneration scaffolds, substantial interest is now growing towards designing multifunctional scaffolds loaded with various molecules and nanomaterials for advanced bone regeneration scaffolds (Figure 7) (Arjunan et al., 2021). For example, in addition to other biofactors and biomolecules, nucleic acids can be added to encode the growth factors that promote bone growth. The discovery of new molecules and bioingredients for manufacturing of future bone generation scaffolds is at the centre of focus and a leading force in studies for bone tissue engineering.

## Conclusion

Fabrication of collagen sponges combined with BG and PGA fibers as a scaffold composite for bone tissue engineering was investigated. *In vitro* studies indicated that addition of BG had a great and impressive effect on both physical and mechanical properties of the composite scaffolds and supported differentiation of MSCs cells towards osteoblasts. The results obtained strongly suggest that addition of the already known bioglass 45S5 component which is FDA approved enhances physical stability of the collagen-PGA scaffold and provides further mechanical strength and biological activity. The scaffold composites reported here, may provide advanced functional properties *in vivo* and have the potential to be considered for additional evaluation towards non-union fracture treatment. Further studies including *in vivo* studies are required to evaluate the biological interactions and functionality of these biodegradable composite scaffolds under real conditions.

## Data Availability

The original contributions presented in the study are included in the article/supplementary material, further inquiries can be directed to the corresponding authors.
